# Iron Surveillance and Management in Gastro-Intestinal Oncology Patients: A National Physician Survey †

**DOI:** 10.3390/curroncol30110714

**Published:** 2023-11-09

**Authors:** Emilie S. Richard, Adriyan Hrycyshyn, Noor Salman, Alliya Remtulla Tharani, Alexandria Abbruzzino, Janet Smith, Jacob J. Kachura, Michelle Sholzberg, Jeffrey D. Mosko, Sami A. Chadi, Ronald L. Burkes, Maya Pankiw, Christine Brezden-Masley

**Affiliations:** 1 Department of Medicine, Mount Sinai Hospital, Sinai Health, Toronto, ON M5G 1X5, Canada; 2 Department of Medicine, Division of Oncology/Hematology, St. Michael’s Hospital, Unity Health Toronto, Toronto, ON M5B 1W8, Canada; 3 Department of Medicine, Temerty Faculty of Medicine, University of Toronto, Toronto, ON M5S 3H2, Canada; 4 Department of Medicine, Division of Gastroenterology, St. Michael’s Hospital, Unity Health Toronto, Toronto, ON M5B 1W8, Canada; 5 Sprott Department of Surgery, Division of General Surgery, Toronto General Hospital, University Health Network, Toronto, ON M5G 2C4, Canada; 6 Department of Surgery, University of Toronto, Toronto, ON M5T 1P5, Canada

**Keywords:** iron deficiency, iron deficiency anemia, anemia, gastrointestinal cancer

## Abstract

Purpose: Iron deficiency (ID) is a complication of gastrointestinal (GI) cancers that may manifest as iron deficiency anemia (IDA). Serum ferritin monitoring and oral iron supplementation have the limitations of being falsely elevated and poorly absorbed, respectively. This study aims to assess the discordance in surveillance, treatment practices, and awareness of ID/IDA in GI cancer patients by Canadian physicians treating these patients. Methods: From February 2020 to September 2021, a 22-question electronic survey was sent to medical oncologists (MOs), surgical oncologists (SOs), and gastroenterologists (GEs). The survey collected information about four domains: physician demographics, surveillance practices, treatment practices, and awareness of ID/IDA in GI cancer patients and ASCO/ASH guidelines. Results: A total of 108 (34 MOs, 19 SOs, and 55 GEs) of the 872 (12.4%) invited physicians completed the survey. Of these, 26.5% of MOs, 36.8% of SOs, and 70.9% of GEs measured baseline iron parameters, with few continuing surveillance throughout treatment. Ferritin was widely measured by MOs (88.9%), SOs (100%), and GEs (91.4%). Iron was supplemented if ID/IDA was identified pre-treatment by 66.7% of MOs, 85.7% of SOs, and 94.2% of GEs. Parenteral iron was prescribed by SOs (100%), while oral iron was prescribed by MOs (83.3%) and GEs (87.9%). Only 18.6% of physicians were aware of the ASCO/ASH guidelines regarding erythropoiesis-stimulating agents with parenteral iron for treating chemotherapy-induced anemia. Conclusion: Results illustrate variations in practice patterns for IDA management across the different physician specialties. Moreover, there appeared to be gaps in the knowledge and care surrounding evidence-based IDA management principles which may contribute to poor clinical outcomes.

## 1. Introduction

Gastrointestinal (GI) cancer refers to malignancies affecting the GI tract, including the stomach, colon, and rectum. In 2022, colorectal cancer (CRC) was estimated to be the second highest cause of cancer mortality, with 24,300 individuals diagnosed and 9400 deaths in the Canadian population [1]. That same year, it was estimated that 4100 Canadians would be diagnosed with gastric cancer, resulting in 2000 deaths [2]. GI cancers are commonly associated with iron deficiency (ID) which may cause iron deficiency anemia (IDA) defined by a decreased total amount of hemoglobin (Hb < 12.0 g/dL) or decreased number of red blood cells (RBC) [3,4,5,6]. ID is classified as absolute or functional iron deficiency (AID or FID); AID occurs when iron reserves are depleted, while FID occurs when there is a lack of biologically available iron due to cancer associated cytokine release [7,8,9]. FID is the predominate mechanism of ID seen in the cancer patient population [3]. The underlying causes of ID in the context of GI cancer include: inadequate dietary intake, malabsorption secondary to tumor interference and surgery, systemic inflammation, select chemotherapeutic agents, and chronic or excessive blood loss from bleeding tumors—all of which synergistically increase the risk of IDA [7,10]. Another important cause of anemia in cancer patients is chemotherapy-induced anemia (CIA), which is defined as the development of acute anemia in relation to myelosuppressive chemotherapy initiation [11,12]. ID, IDA, and CIA are frequent complications of cancer and cancer therapy, both at diagnosis and during treatment [6,8]. Common symptoms of anemia include: fatigue, weakness, impaired physical performance, diminished cognitive function, and decreased quality of life (QoL) [3,4,6]. Importantly, anemia is associated with an increased risk of mortality in cancer patients [13,14,15]. Prompt recognition and management of anemia throughout treatment has been linked to improved clinical outcomes and better tolerance to anti-cancer therapies [7,15].

Common lab tests for ID surveillance include ferritin, serum iron, total iron binding capacity (TIBC), and transferrin saturation (TSAT). Cancer patients with FID can be identified with TSAT < 20% and serum ferritin ≥ 100 ng/mL [9]. Recognizing ID/IDA in the GI oncology population presents a challenge. While serum ferritin is an intracellular iron storage protein that accurately tests to diagnose ID, it is also an acute phase reactant and can be falsely elevated in cancer patients [4,8,10]. It is well known that ferritin can be elevated in patients with chronic inflammation (as a result of chemotherapy and/or active cancer) or infection and its diagnostic applicability in these clinical settings is considerably impaired. In this situation, TSAT is more reliable for diagnostic purposes, although is rarely used in this context [5,8,9].

Methods of ID treatment include parenteral iron, oral iron, and RBC transfusion. Parenteral iron (e.g., ferric carboxymaltose) is an intravenous (IV) supplementation that is a safe and effective treatment option for the management of ID/IDA [16,17,18]. The effectiveness of oral iron (e.g., ferrous sulphate) in GI cancer patients may be limited because of malabsorption of iron in the intestine, trapped iron within enterocytes due to metabolic disorders induced by inflammatory cytokines, lack of patient adherence because of GI side effects, and longer treatment time needed to increase Hb levels [8,9,19]. RBC transfusions increase Hb and iron levels to treat severe anemia, but can be associated with increased morbidity and mortality [11,20]. Another method for iron repletion is the use of erythropoiesis stimulating agents (ESAs), which are synthetic proteins used to increase the process of making RBCs. ESAs effectively increase Hb levels without the need for RBC transfusions; however, they are expensive and have potentially toxic side effects. Various clinical trials have looked at the effects of using both IV iron and ESAs together, and have seen multiple benefits surrounding hematopoietic response, improved Hb levels, lower risk of RBC transfusions, faster response time, improved QoL, and little to no adverse events [7,9,21,22]. The American Society of Clinical Oncology/American Society of Hematology (ASCO/ASH) guidelines recommend the use of ESAs for patients with CIA whose cancer treatment does not have a curative intent, and whose Hb levels are <10 g/dL. Additionally, the guidelines recommend RBC transfusion as a viable option [23].

We proposed a Canada-wide electronic survey sent to medical oncologists (MOs), surgical oncologists (SOs), and gastroenterologists (GEs) who treat luminal GI malignancies. The purpose of this study was to better understand the practices in ID/IDA management throughout treatment, the beliefs and attitudes toward iron monitoring and supplementation, and Canadian physicians’ awareness concerning iron surveillance and supplementation methods, as it relates to clinical outcomes in this patient population. We hypothesize a wide disparity in the surveillance, treatment practices, and awareness of ID/IDA for this patient population. 

## 2. Methods

### 2.1. Study Design and Population

This Mount Sinai Hospital Research Ethics Board (protocol code 20-0056-E and approval date: 14 May 2020) approved, cross-sectional study involved a 22-question electronic survey disseminated to 872 eligible physicians (an MO, SO, or GE who was involved in the management of patients with GI malignancies (gastric or colorectal) across Canada). The eligible physicians were conveniently sampled and classified into one of three subgroups: MOs, SOs, and GEs. An invitation to complete the survey was emailed to all eligible physicians, and the email contained a link to an online survey developed using the software Survey Monkey. The data collection period occurred from 20 February 2020 until 19 September 2021. General reminder emails were sent on two occasions to maximize response rate. Participation in the survey was completely voluntary. Responding to the questions (completely or partially) would imply consent. 

### 2.2. Data Collection and Analysis

The four main domains of questions in the survey included demographics, surveillance practices, treatment practices, and physician awareness. The demographic variables collected about the participants were specialty, experience, location, and gender. Iron surveillance practices analyzed measurements of ferritin, serum iron, TIBC, and iron saturation (see Appendix A for glossary of terms). In addition, treatment practices were assessed using the supplementation methods of parenteral iron, oral iron, and RBC transfusion. Lastly, physicians were asked about their awareness of ID/IDA in GI oncology patients and current ASCO/ASH guidelines. 

Data analysis was conducted for this cross-sectional observational study. The physician characteristics, iron surveillance, iron supplementation, and awareness of the ASCO/ASH guidelines were summarized as counts and percentages or means and standard deviations (SD). A mean percentage of patients that presented with ID/IDA was determined by a subjective estimate of the average number of GI oncology patients presenting with ID/IDA as reported by the physicians divided by the sum of physicians that responded. The counts, percentages, means, and SD values were calculated using the application Excel.

## 3. Results

### 3.1. Physician Characteristics

A total of 108 (34 MOs, 19 SOs, and 55 GEs) of the 872 invited physicians completed the survey, resulting in a 12.4% response rate. The majority of physicians were from Ontario with 24 (70.6%) MOs, 15 (78.9%) SOs, and 32 (59.3%) GEs (Table 1). There were no physician specialties represented from five provinces/territories in this study (NB, PE, NU, YU, NT). The institution type with the most physicians was academic/teaching with 27 (79.4%) MOs, 12 (63.2%) SOs and 37 (68.5%) GEs. The mean number of years in practice was 13.2 ± 11.0 (MOs), 14.0 ± 10.2 (SOs) and 13.4 ± 8.9 (GEs). A total of 6 (11%) GEs and 5 (26.3%) SOs saw more than 10 GI cancer patients per month compared to 23 (67.6%) MOs. Overall, the majority of physicians were from Ontario (*n* = 71, 65.7%) and practicing in academic/teaching institutions (*n* = 76, 70.4%) with a wide range of years in practice.

### 3.2. Surveillance Practices

**Figure 1:**
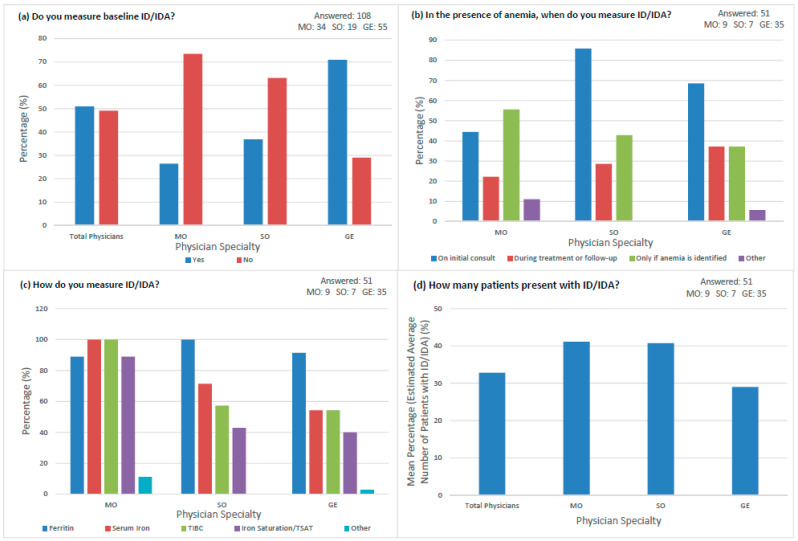
Iron surveillance practices by physician specialty. (**a**) Proportion of physicians that measure baseline iron parameters for GI oncology patients. (**b**) Time of iron parameter measurement in the presence of anemia by specialty. (**c**) Lab tests for ID assessment ordered by specialty. (**d**) Mean percentage of IDA patients observed in the GI oncology patient population by specialty.

All physicians (*n* = 108) responded to the question assessing whether they measured baseline iron parameters for their GI oncology patients. The total physician population was nearly evenly split with 50.9% (*n* = 55) measuring baseline iron parameters (Figure 1a). A greater proportion of GEs (*n* = 39, 70.9%) compared to SOs (*n* = 7, 36.8%), and MOs (*n* = 9, 26.5%) measured baseline iron parameters. Of the 55 physicians who measured baseline iron parameters, 51 physicians (9 MOs, 7 SOs, and 35 GEs) consistently answered the subsequent iron surveillance questions in Figure 1. Iron parameters were largely measured at initial consult by MOs (*n* = 4, 44.4%), SOs (*n* = 6, 85.7%), and GEs (*n* = 24, 68.6%), with few continuing surveillance throughout treatment course (Figure 1b). For ID assessment, 32 (91.4%) GEs and 7 (100%) SOs relied on ferritin to measure iron parameters, while MOs were evenly distributed in their evaluation of ferritin (*n* = 8, 88.9%), serum iron (*n* = 9, 100%), TIBC (*n* = 9, 100%), and iron saturation (*n* = 8, 88.9%) (Figure 1c). When asked what percentage of GI patients presented with IDA, MOs and SOs stated similar mean percentages (41.1% and 40.7%, respectively), while GEs stated a lower mean percentage (29%) of their patient population. A subjective estimate of the average number of GI oncology patients presenting with ID/IDA (the mean percentage) reported by 51 physicians was 32.7% ± 19.8 (Figure 1d). There were variations in the choice of tests physicians used to measure iron parameters amongst and within specialty types. 

### 3.3. Treatment Practices

**Figure 2:**
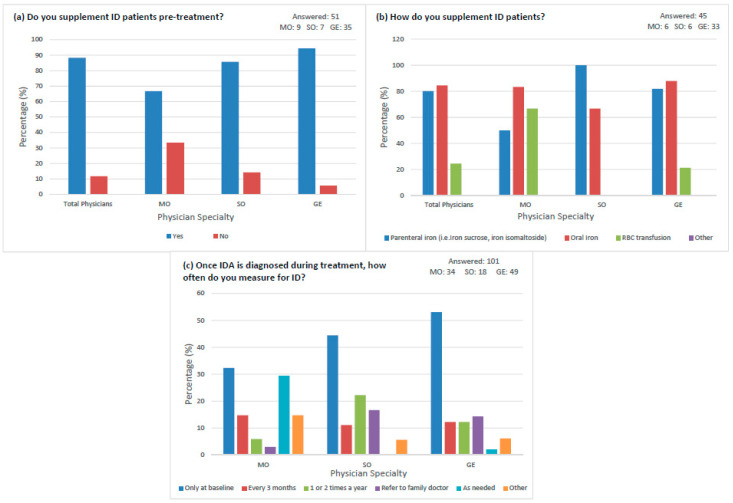
Treatment practices for iron supplementation by physician specialty. (**a**) GI oncology patients with ID that receive pre-treatment supplementation by specialty. (**b**) Type of iron supplementation for ID patients preferred by specialty. (**c**) Frequency of ID measurement during treatment in anemic GI oncology patients by specialty.

Various treatment practices for iron supplementation based on specialty were also assessed. The majority of physicians (88.2%, *n* = 45) supplemented iron if ID/IDA was identified prior to systemic/surgical oncologic treatment, including 66.7% (*n* = 6) of MOs, 85.7% (*n* = 6) of SOs, and 94.3% (*n* = 33) of GEs (Figure 2a). When asked how physicians supplement ID patients, physicians could select more than one treatment choice. Parenteral iron was the preferred treatment choice for SOs (*n* = 6, 100%), while oral iron was preferred among GEs (*n* = 29, 87.9%) and MOs (*n* = 5, 83.3%) (Figure 2b). Out of the GI oncology patients that were anemic, most physicians (32.4% of MOs, 44.4% of SOs, and 53.1% of GEs) only screened at baseline when compared to other time points during treatment. Screening anemic patients as needed during treatment was observed by 29.4% of MOs, compared to 2.0% of GEs, and none of the SOs (Figure 2c). 

### 3.4. Physician Awareness

**Figure 3:**
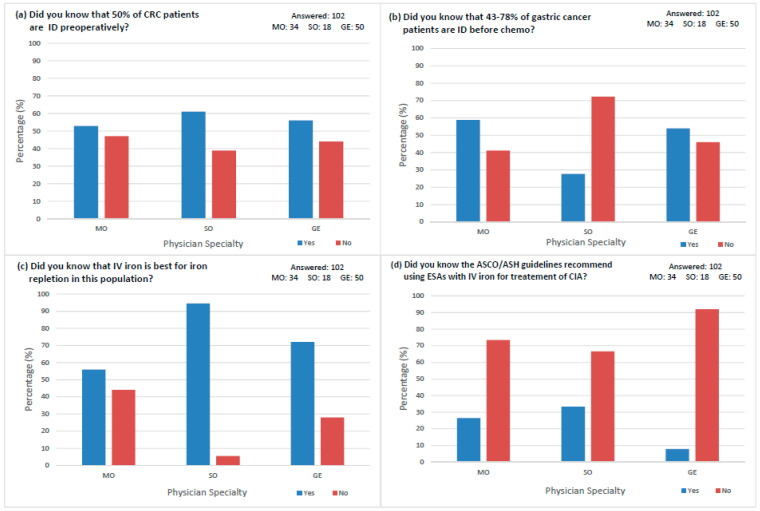
Physician Awareness of ID/IDA in GI Oncology Patients and ASCO/ASH Clinical Practice Guideline by Physician Specialty. (**a**) Awareness that close to 50% of CRC patients are iron deficient preoperatively. (**b**) Awareness that 43–78% of gastric cancer patients are iron deficient prior to starting chemotherapy. (**c**) Awareness that IV iron supplementation is the best method for iron repletion in this population. (**d**) Awareness of the recent ASCO/ASH Clinical Practice Guideline regarding the use of ESAs in conjunction with parenteral iron supplementation for treatment of CIA.

A series of physician awareness questions were asked, and 102 physicians responded. About half of all physicians surveyed were unaware that close to 50% of CRC patients have ID preoperatively, including 47.1% (*n* = 16) of MOs, 38.9% (*n* = 7) SOs, and 44% (*n* = 22) of GEs (Figure 3a). When asked if they knew that 43–78% of gastric cancer patients have ID prior to starting chemotherapy, about half of MOs and GEs were unaware (41.2% and 46.0%, respectively), while most SOs were unaware (72.2%) (Figure 3b). Most physicians were aware that IV parenteral iron supplementation is the best iron repletion method in this population, including 55.8% (*n* = 19) of MOs, 94.4% (*n* = 17) of SOs, and 72% (*n* = 36) of GEs (Figure 3c). When asked about the current ASCO/ASH guidelines regarding the use of ESAs in conjunction with parenteral iron supplementation for treating anemia, 73.5% (*n* = 25) of MOs, 66.7% (*n* = 12) of SOs, and 92% (*n* = 46) of GEs were unaware of these guidelines (Figure 3d). 

## 4. Discussion

This cross-sectional study is the first and largest study evaluating multispecialty clinician practices relating to absolute and functional iron deficiency anemia management in patients with GI cancer. This study is important as IDA is entirely correctable and associated with improvements in both health related QoL and patient performance which ultimately affects survival. In this cross-sectional study, the national survey results presented evidence suggesting the existence of inconsistencies in practice patterns between different specialties regarding ID/IDA surveillance and treatment in GI oncology patients. Management of ID/IDA is clinically relevant to all GI cancer patients, and should be incorporated into routine care for each patient. To create the most comprehensive and standardized care for all GI cancer patients, learning from survey results such as this one can aid in pointing healthcare practitioners and educators in the correct direction, even if these results are only reflective of a certain subset of clinicians.

Our study had some weaknesses that should be considered. We recognize that each physician specialty type and institution of practice were not represented in equal proportion in this study. Additionally, not having physicians represented from each province and territory in the study means our results may not be reflective of practices on a national scale. Furthermore, the low response rate (12.4%) to the survey may also reflect the significant need to educate and improve awareness of ID/IDA and treatment guidelines in oncology. These survey results of 12.4% of physicians involved in GI cancer patient management, may also have been a self-selection-biased cohort as these physicians were interested in and aware of testing for ID. Additionally, not all 12.4% of physicians responded to every question in the survey. For instance, approximately only half of the 12.4% of physicians provided a subjective estimate of the average number of GI oncology patients presenting with ID/IDA in Figure 1d, which may hinder the reliability of results. The lower response rate to certain survey questions enforces the need to increase physician awareness of ID/IDA treatment guidelines and encourage physicians to be proactive in future research studies to improve the response rate and reliability of results. Another limitation of the survey was not asking the reason why some physicians did not screen for anemia prior to and during cancer treatment, and not investigating the reason for observed preferences in choice of ID/IDA treatment. Future research examining the reasons why physicians selected certain surveillance and treatment methods is recommended to gain a more profound understanding of the observed practices relating to ID/IDA management in GI cancer patients. 

### 4.1. Lack of Adequate Iron Surveillance 

Our data shows that the majority of MOs (73.5%) and SOs (63.2%) did not measure baseline iron parameters. We observed that these physicians also did not respond to questions regarding the time points and frequency of iron surveillance, suggesting that most respondents do not include iron surveillance in their routine practice. Results revealed that even when iron parameters were measured, it was not always continued during the course of treatment. Repeated testing of iron parameters during treatment is crucial to ensure adequate iron repletion in patients with persistent anemia, or who have undergone surgeries–such as a gastrectomy–causing chronic ID due to malabsorption [7,24]. The most reliable ID diagnostic test is TSAT for this patient population; however, SOs and GEs used it the least (42.9% and 40%, respectively). Ferritin appeared to be the most widely used test across all physician types despite its diagnostic limitation of being falsely elevated in cancer patients by acting as an acute phase reactant [4,8,10]. Our study results regarding surveillance practices may be compared to a cross-sectional study observing management of anemia and iron levels in CIA patients across Europe. This study revealed that iron levels were predominantly assessed using Hb (94%) and ferritin (48%), with limited testing using TSAT (14%) [25]. Similarly to our study, these results suggest that TSAT parameters were less frequently measured by physicians, while ferritin levels were more regularly monitored despite being less reliable. The same study also revealed that the management of anemia and iron status in patients with CIA varied substantially between different physicians, reinforcing the need for guidelines establishing iron surveillance practices most suitable for GI oncology patients. In our study, the participating physicians estimated that about one-third of their GI oncology patients presented with ID/IDA. It remains to be explored why many physicians do not adequately perform iron surveillance even though many of their patients are anemic and/or iron deficient. 

### 4.2. Lack of Evidence-Based IDA Treatment 

Of the physicians who measured baseline ID/IDA (Figure 1a), most physicians (88.2%) supplemented ID patients pre-treatment. This aligns with the recommendation that iron supplementation should be administered pre-treatment before surgery [26]. The survey results showed that although the majority of physicians used the most effective treatment method of parenteral (IV) iron, they also largely prescribed oral iron which may not be necessary or effective. The benefits of IV iron can be demonstrated in a meta-analysis of randomized controlled trials, where IV iron was demonstrated to decrease the percentage of blood transfusions required while increasing the hematopoietic response when compared to oral iron administered to cancer patients with CIA [27]. Oral iron supplements, provided as ferrous or ferric salts, are ineffective in cancer patients due to malabsorption from absorptive inflammatory blockage within enterocytes and reduced intestinal iron absorption in these patients, causing more than 95% of this element to be excreted from the body. Additionally, there may be a decreased adherence to oral iron treatment due to GI side effects, such as constipation, abdominal pain, bloating, and nausea [8,9,19]. An innovative oral iron formulation called sucrosomial iron (SI) consisting of ferric pyrophosphate protected by a phospholipid bilayer and a sucrester matrix (sucrosome) has demonstrated similar effectiveness, with lower risks and lower cost when compared to IV iron. In several small case series and studies of oncology patients with ID/IDA treated with oral SI (30–60 mg/day for 2–6 months between 2015 and 2017), there was an overall increase in Hb levels, with few GI side effects [28,29]. Although parenteral (IV) iron and oral SI treatment demonstrate promising results in the treatment of ID/IDA in GI cancer patients, there is not one correct overarching treatment method, and the physicians should modify the treatment choice and dosage based on severity of ID/IDA (mild, moderate, or severe). Formal guidelines with respect to treatment selection and dosage requirements should be established to ensure physicians administer the most effective course of treatment based on the severity of ID/IDA in this patient population. From our data, there appeared to be a pattern of only testing at baseline and not following through during the course of treatment, even in anemic GI oncology patients. This practice is contrary to the ASH hematology guideline which recommends screening every 3–6 months after initial iron repletion treatment to determine if ongoing iron supplementation is required [30]. Following the ASCO/ASH guidelines is particularly important in GI oncology patients, as it reduces treatment-induced adverse events. It is also in line with current Choosing Wisely campaigns to decrease RBC transfusions when patients are anemic if safer non-transfusion options are available [31]. Our results indicated that although most physicians supplemented for ID/IDA pre-treatment as per recommendations, many physicians did not provide the most effective supplementation method for the GI oncology patient population. Additionally, our data revealed that even when the guidelines existed about monitoring iron levels during treatment, they were not always followed. 

### 4.3. Self-Selection Bias 

The clinicians who participated in this study are likely more invested in the management of IDA, representing self-selection bias. Thus, it is likely that these findings are attenuated which suggest an even larger knowledge gap amongst other practicing clinicians managing this patient population. Given this low response rate, drawing concrete conclusions about the prevalence of management of ID/IDA in gastric cancer patients is not possible. However, taking the population that provided responses to the survey under consideration, it is evident that there is lack of physician awareness in at least this subset of physicians. Thus, extrapolating this data to reach the broader physician population can yield the assumption that there is a general lack of ID/IDA management amongst Canadian physicians. 

### 4.4. Lack of Physician Awareness

We found that about half of physicians who did not measure baseline iron parameters were unaware of the extent of ID/IDA in the GI oncology patient population. This lack of awareness could be a possible explanation for the absence of screening practices (Figure 1a and Figure 3a,b). Around half of the MOs and GEs were not aware that 50% of CRC patients and 43–78% of gastric cancer patients present with ID pre-treatment [32,33]. The importance of physician awareness of ID/IDA in CRC patients can be compared to a literature review discussing IDA as a comorbidity of medical conditions such as cancer, in which increased awareness about the prevalence of IDA helped with the early detection and management of anemia [34]. Most physicians (Figure 3c) were aware that IV iron is the best method for iron repletion in this population [35]. We observed that SO were the most aware that IV iron is best for iron repletion and used IV iron the most, while MO were the least aware and used IV iron the least (Figure 2b and Figure 3c). Increased awareness in SOs is based on data demonstrating that, if IV iron repletion occurs pre-operatively, morbidity and mortality could potentially improve [17]. Our data highlighted that the vast majority of physicians (Figure 3d) did not know the ASCO/ASH guidelines regarding the use of ESAs in conjunction with IV iron supplementation for treating anemia in the GI oncology patient population, specifically when CIA occurs [23]. This data illustrated that, not only were physicians unaware of ID/IDA in their patient population, they were also not up-to-date with ASCO/ASH Clinical Practice Guidelines. Filling in this knowledge gap could prove to be beneficial for patients in terms of improved response to therapy and overall QoL. Our study’s results may be compared to a cross-sectional survey study conducted by ASCO’s Geriatric Oncology Task Force with the objective of assessing practice patterns and barriers to geriatric assessment. The study concluded that only 52% of care providers for older adults were aware of the ASCO Guidelines [36]. Although the development of guidelines is essential, there must also be increased education and awareness of the developed guidelines to ensure they are followed by physicians. Another important consideration is the access to treatment with IV iron repletion. The consensus guidelines should discuss access to IV iron, availability of infusion clinics, nursing staff administering IV iron, and insurance coverage of IV iron. Therefore, building on this data provides the framework for future work through guideline development to ensure that identification of ID and rapid iron repletion occurs, especially if patients require surgery or myelosuppressive chemotherapy for their cancer treatment.

The overall results of our survey can be compared to a recent cross-sectional study analyzing the practices around patient blood management (PBM) of Dutch coloproctological surgeons in 2017 [37]. The study found a wide range of iron surveillance and repletion practices both within institutions and across the country likely due to a lack of guidelines set in place for proper management of CRC patients. For instance, half of hospitals (49.3%) lacked clear policies for managing mild–moderate preoperative anemia. To improve effectiveness and uniformity of PBM, improving education, establishing evidence-based treatment guidelines, and appointing key leaders to manage PBM projects was recommended [35]. The need for PBM improvements was addressed in Canada, as the National Advisor Committee (NAC) on Blood and Blood Products published guidelines and recommendations in 2022 for successful PBM programs, highlighting aspects such as education, required resources, and funding [38]. Our study also suggests an increased need for anemia treatment guidelines as we observed a similar lack of consensus in practices amongst Canadian physicians who are involved in the management of ID/IDA in GI cancer patients. ID/IDA appeared to be under acknowledged in GI oncology populations and may have contributed to worsened symptomology and clinical outcomes for many patients. The data collected thus far reveal a potential knowledge gap worthy of further investigation, and supports the need for a national consensus guideline addressing variations in GI-cancer IDA surveillance and care. A limitation of this survey was not asking whether prescribing physicians had access to infusion centers or parenteral iron causing barriers to treatment; therefore, guideline development would potentially provide support regarding access to care. As iron deficiency is the most common hematological complication in individuals with CRC, the research revealed by this survey study along with future research in this field may be applied in the development of standardized guidelines in the interest of improving the QoL and treatment response of ID/IDA cancer patients. Establishing a national consensus guideline and increasing education regarding effective surveillance and treatment methods for ID/IDA could improve the care of GI oncology patients in Canada.

## Figures and Tables

**Table 1:** Physician Characteristics.

	Medical Oncologists *n* = 34	Surgical Oncologists *n* = 19	Gastroenterologists *n* = 55
Gender, Male	19 (55.9%)	8 (42.1%)	38 (69.1%)
Years of Practice (mean ± SD)	13.2 ± 11.0	14.0 ± 10.2	13.4 ± 8.9
0–5	9 (28.1%)	4 (21.1%)	10 (19.2%)
6–10	6 (18.8%)	5 (26.3%)	14 (26.9%)
11–20	11 (34.4%)	7 (36.8%)	18 (34.6%)
>20	6 (18.8%)	3 (15.8%)	10 (19.2%)
Province of Practice			
ON	24 (70.6%)	15 (78.9%)	32 (59.3%)
QC	3 (8.8%)	1 (5.3%)	3 (5.6%)
NS	2 (5.9%)	0 (0.0%)	2 (3.7%)
NB	0 (0.0%)	0 (0.0%)	0 (0.0%)
NL	0 (0.0%)	1 (5.3%)	1 (1.9%)
PE	0 (0.0%)	0 (0.0%)	0 (0.0%)
MB	2 (5.9%)	1 (5.3%)	4 (7.4%)
AB	1 (2.9%)	0 (0.0%)	8 (14.8%)
SK	0 (0.0%)	1 (5.3%)	0 (0.0%)
BC	2 (5.9%)	0 (0.0%)	4 (7.4%)
NU	0 (0.0%)	0 (0.0%)	0 (0.0%)
YT	0 (0.0%)	0 (0.0%)	0 (0.0%)
NT	0 (0.0%)	0 (0.0%)	0 (0.0%)
Institution Type			
Academic/Teaching	27 (79.4%)	12 (63.2%)	37 (68.5%)
Community	7 (20.6%)	7 (36.8%)	16 (29.6%)
Private	0 (0.0%)	0 (0.0%)	1 (1.9%)
Number of luminal GI tumor patients/month (mean ± SD) ^+^	33.9 ± 36.0	12.1 ± 40.3	16.8 ± 28.7
0–5	5 (14.7%)	11 (57.9%)	34 (61.8%)
6–10	6 (17.6%)	3 (15.8%)	15 (27.3%)
11–20	7 (20.6%)	2 (10.5%)	3 (5.5%)
21–40	7 (20.6%)	1 (5.3%)	2 (3.6%)
41–60	4 (11.8%)	1 (5.3%)	0 (0.0%)
60+	5 (14.7%)	1 (5.3%)	1 (1.8%)

^+^ Luminal GI tumors include esophageal, gastric, small bowel, and colorectal cancers. Note: Data for each characteristic are presented as frequency (percent) unless otherwise specified.

## Data Availability

The data presented in this study are available on request from the corresponding author. The data are not publicly available due to privacy or ethical restrictions.

## Appendix A

**Table A1:** Glossary of Key Terms.

Term	Definition
Absolute iron deficiency (AID)	Iron deficiency that occurs when iron reserves are depleted.
American Society of Clinical Oncology/American Society of Hematology (ASCO/ASH)	Societies that establish guidelines with recommendations of treatment and care methods for clinicians.
Chemotherapy-induced anemia (CIA)	Development of acute anemia in relation to initiation of myelosuppressive chemotherapy.
Colorectal cancer (CRC)	Type of cancer affecting the colon and/or rectum.
Erythropoiesis stimulating agents (ESA)	Synthetic proteins used to increase the production of red blood cells.
Functional iron deficiency (FID)	Iron deficiency that occurs when there is a lack of biologically available iron due to cancer associated cytokine release.
Gastroenterologists (GE)	Physicians trained to diagnose and treat problems in the gastrointestinal tract and liver.
Gastrointestinal (GI)	The gastrointestinal tract refers to the stomach, colon, and rectum.
Hemoglobin (Hb)	Protein located in red blood cells responsible for carrying oxygen.
Intravenous (IV)	Medical technique used to inject formulated fluids directly into a vein.
Iron deficiency (ID)	Decrease in total iron content in the body.
Iron deficiency anemia (IDA)	Iron deficiency defined by a decreased total amount of hemoglobin or decreased number of red blood cells.
Medical oncologists (MO)	Physicians specialized in the diagnoses, treatment, and prevention of cancer.
Quality of Life (QoL)	An individual’s perception of their overall well-being assessed in the context of personal health in this study.
Red blood cells (RBC)	Cells that carry oxygen from the lungs to be delivered all over the body.
Red blood cell (RBC) transfusion	Procedure involving the donation of blood through an intravenous line.
Standard Deviation (SD)	Measure demonstrating the amount of variation from a mean.
Sucrosomial iron (SI)	Innovative method of oral iron supplementation in which ferric pyrophosphate is protected by a phospholipid bilayer and a sucrester matrix (sucrosome).
Surgical oncologists (SO)	Physicians focused on the treatment of cancer using surgical methods.
Total iron binding capacity (TIBC)	Blood test used to measure the ability of blood to bind to iron and transport it throughout the body.
Transferrin saturation (TSAT)	Ratio of serum iron to total iron binding capacity. Also referred to as iron saturation.
